# Supplementary material for: Electric transmission value and its drivers in United States power markets
Source: Nat Commun. 2025 Aug 28;16:8055. doi: 10.1038/s41467-025-63143-5 (PMC12394553; doi:10.1038/s41467-025-63143-5)
Supplement: Supplementary file 1 — Supplementary Information [file 41467_2025_63143_MOESM1_ESM.pdf]

Supplementary Information

Geospatial patterns of transmission value (day-ahead)

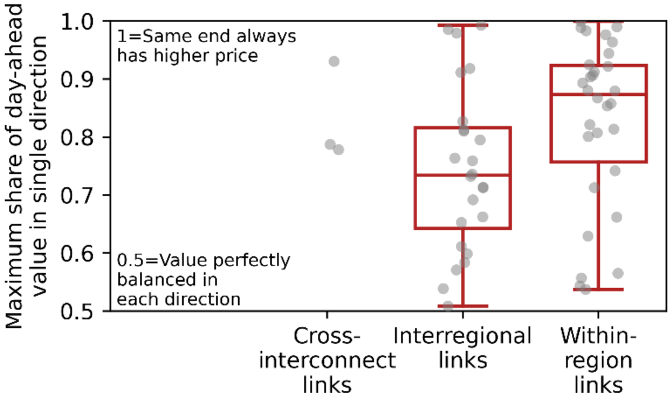

Supplementary Figure 1 Consistency of high-low price direction (day-ahead market). Each point represents one of the 57 links. Links connected to the non-ISO West where there is not a day-ahead market are excluded. The horizontal lines on each box plot show, from low to high, the smallest data point lying within 1.5x the inter-quartile range (IQR) from the 25th percentile, the 25th percentile, the 50th percentile (median), the 75th percentile, and the largest data point lying within 1.5x the IQR from the 75th percentile.

Temporal trends in transmission value (day-ahead)

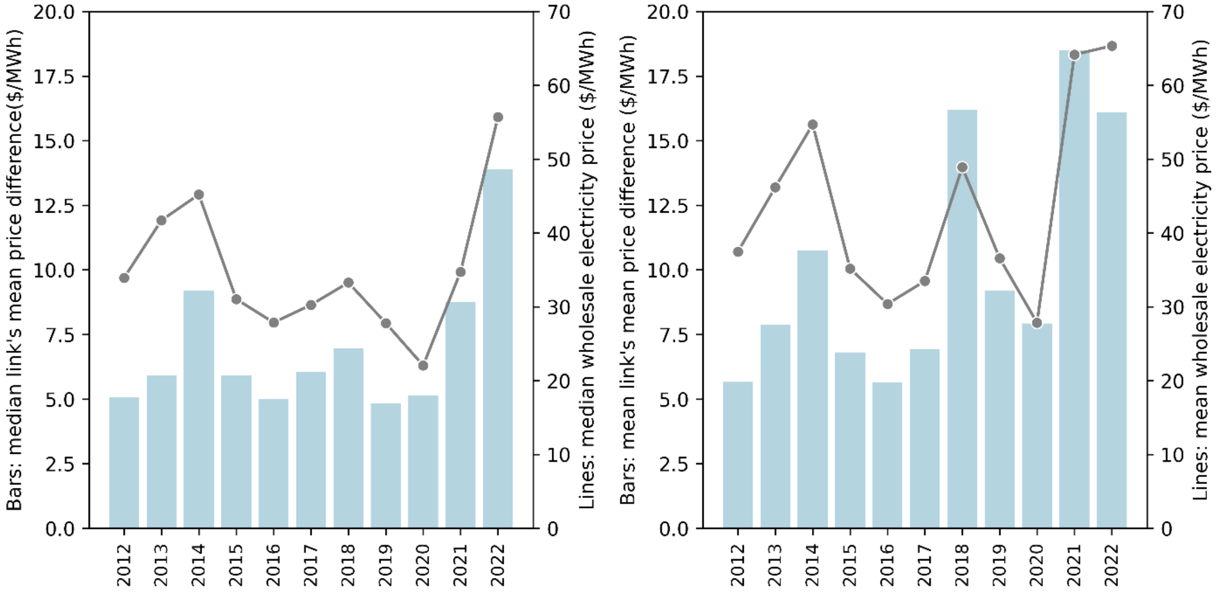

Supplementary Figure 2 Median (left) and mean (right) day-ahead market value of transmission (bars) and wholesale electricity price (lines) across the set of 57 links. Links connected to the non-ISO West where there is not a day-ahead market are excluded. Note that the set of links in the early years is smaller due to data constraints, as explained in the Methods.

Conditions during times of peak transmission value

Hot weather was found to have a limited relationship with transmission value and typically impacts peak transmission value periods by driving high net load. Of the hottest 5% of days in each location, 7% of hours overlap with the peak value hours suggesting that a hot weather hour is only 2 percentage points more likely to experience peak transmission value than an hour picked at random. However, the hottest 1% of days in each location show a stronger relationship with transmission value: 10% of hours on these days overlap with peak value hours. Nearly all of these high-heat peak-value hours and their associated transmission value overlap with unforeseen events, high renewable generation, or high net load conditions (89% of hours, 95% of value). Because including hot weather as a stand-alone condition would only decrease the proportion of transmission value labeled “None of the Above” in Figure 6a by 0.001, we do not focus on it in our results.

Some of the peak value occurs during specific events known to have impacted the electricity grid, but most is found outside of these extreme events. We find that these designated events, defined as key weather events identified in the literature (e.g., named storms, heatwaves) or as periods of grid stress identified by the North American Electric Reliability Corporation (NERC), are present for 11% of peak value hours, representing 21% of total value during peak value hours. See Supplementary Table *2* for a list of designated events. The impact of these events is generally already captured by the other underlying conditions discussed in this section, to the extent that including these designated events would only decrease the proportion of transmission value labeled “None of the Above” in Figure 6a by 0.003. Such a result suggests that transmission value is driven by more routine instances of uncertainty and cold snaps, rather than by historic reliability events documented by NERC or caused by memorable storms.

	**Percentage of time condition overlaps with peak transmission value, where peak is defined as the top __ of hours**			**Percent of all hours**
	**1%**	**5%**	**10%**	
**Unforeseen intraday variance**	12.9%	43.2%	58.1%	6.7%
**High net load** (highest 5%)	4.1%	13.3%	22.0%	5.7%
**Cold weather** (coldest 5%)	3.7%	13.1%	21.3%	6.2%
**Hot weather** (hottest 5%)	1.9%	7.3%	13.1%	7.0%
**Hot weather** (hottest 1%)	2.9%	9.9%	17.0%	1.6%
**High renewable generation** (highest 5%)	1.4%	9.5%	20.4%	6.3%
**Designated events**	3.4%	10.6%	17.7%	5.3%

Supplementary Table 1 Summary of analyzed system conditions: prevalence and impact on peak transmission value (real-time). Aggregate results for all 52 studied links within or between ISO or RTO regions, excluding those in the non-ISO West and Southeast.

Geographic differences in conditions during times of peak transmission value – two case studies

Between PJM_VA and MISO_INHub there is an average price difference of $14/MWh, equivalent to an average marginal value of $125 million per year for a 1 GW link during the 11-year study horizon. At least one of the four key conditions is present for over 96% of peak transmission value. At 81%, unexpected events are the most prevalent condition, but most unexpected events occur at the same time as at least one of the other conditions. The coldest days correspond to 45% of the peak value and regularly include high net load hours, presumably driven by heating demand. 11% of peak value occurs when renewable

generation is at its highest levels; typically there is also an unexpected event during these hours. Supplemental Supplementary Figure 3 depicts these findings.

Within SPP, an east-west link across Kansas has an average price difference of $20/MWh, equivalent to an average marginal value of $174 million per year for a 1 GW link over the 11-year study horizon. Here, unexpected events and high renewable generation are the key conditions present during peak transmission value periods. An unexpected event occurred during 77% of peak transmission value, with limited overlap with other studied conditions. High renewable generation, present for 20% of peak value, is the second-most prevalent condition. Supplemental Supplementary Figure 4 depicts these findings. There is a persistent pricing gradient between these nodes, with the real-time price at the eastern node higher than that at the western node for 82% of the studied hours and 91% of the transmission value. When renewable generation in SPP is near its peak (in the top 5%), the direction of the pricing differential is even more predictable: the price is higher in the east for 97% of the hours and 99% of the transmission value. This pattern is consistent with the high penetration of wind generation in western SPP depressing prices there (median price of -$15.62 during high renewable generation versus $18.55 overall) while prices on the eastern side of Kansas are above typical levels (median price of $33.26 during high renewable generation versus $24.67 overall) despite the surplus of low marginal cost generation on the other side of the transmission constraints.

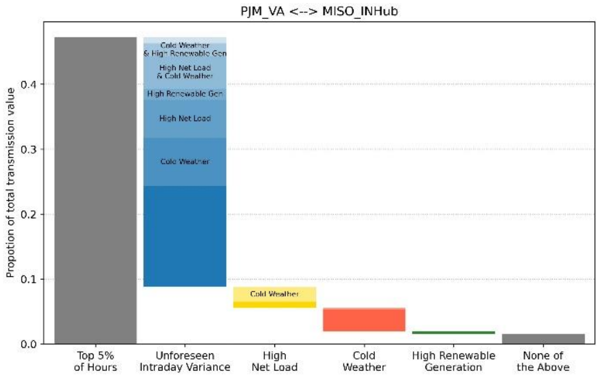

Supplementary Figure 3 Contribution of key system conditions to the marginal transmission market value during peak hours of a link between PJM's Dominion Hub and MISO's Indiana Hub.

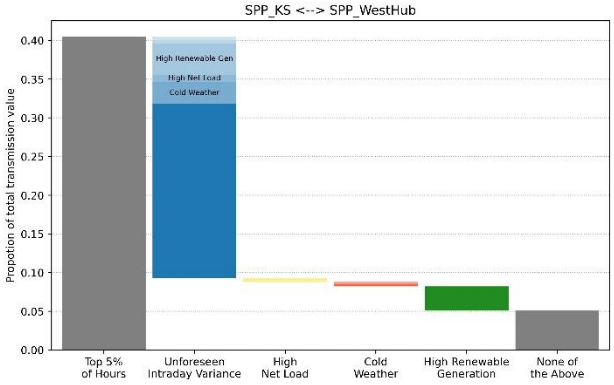

Supplementary Figure 4 Contribution of key system conditions to the marginal transmission market value during peak hours of an east-west link across Kansas.

Market depth and saturation effects

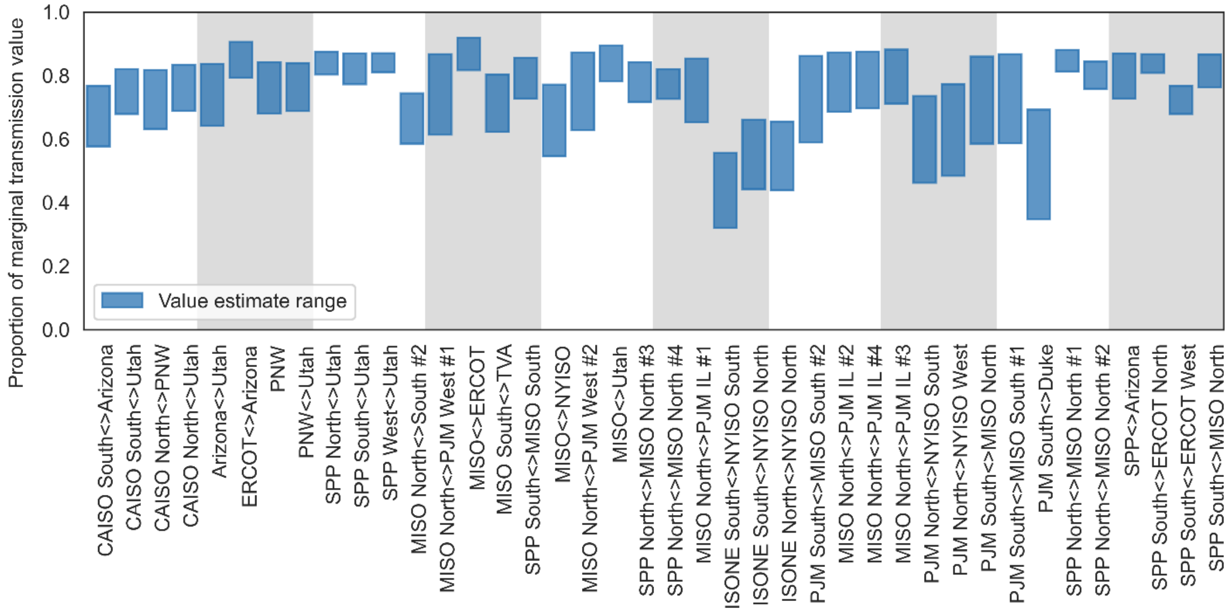

Supplementary Figure 5 Range of transmission market value estimates accounting for market depth of a 1 GW transfer capacity increase, when <u>assuming the relevant market size is 50% of the size used in the main paper</u>, relative to marginal transmission value (real-time). Grey and white bands are used to improve readability; they do not communicate information about the results.

Transmission costs compared to market value

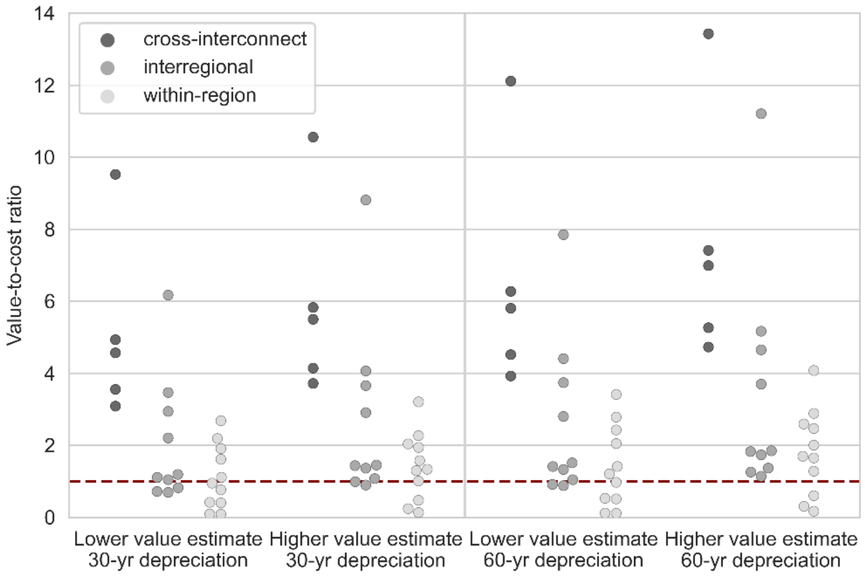

Supplementary Figure 6 Comparison of project costs to market value estimates that account for market depth under different choices of value estimate and depreciation rate.

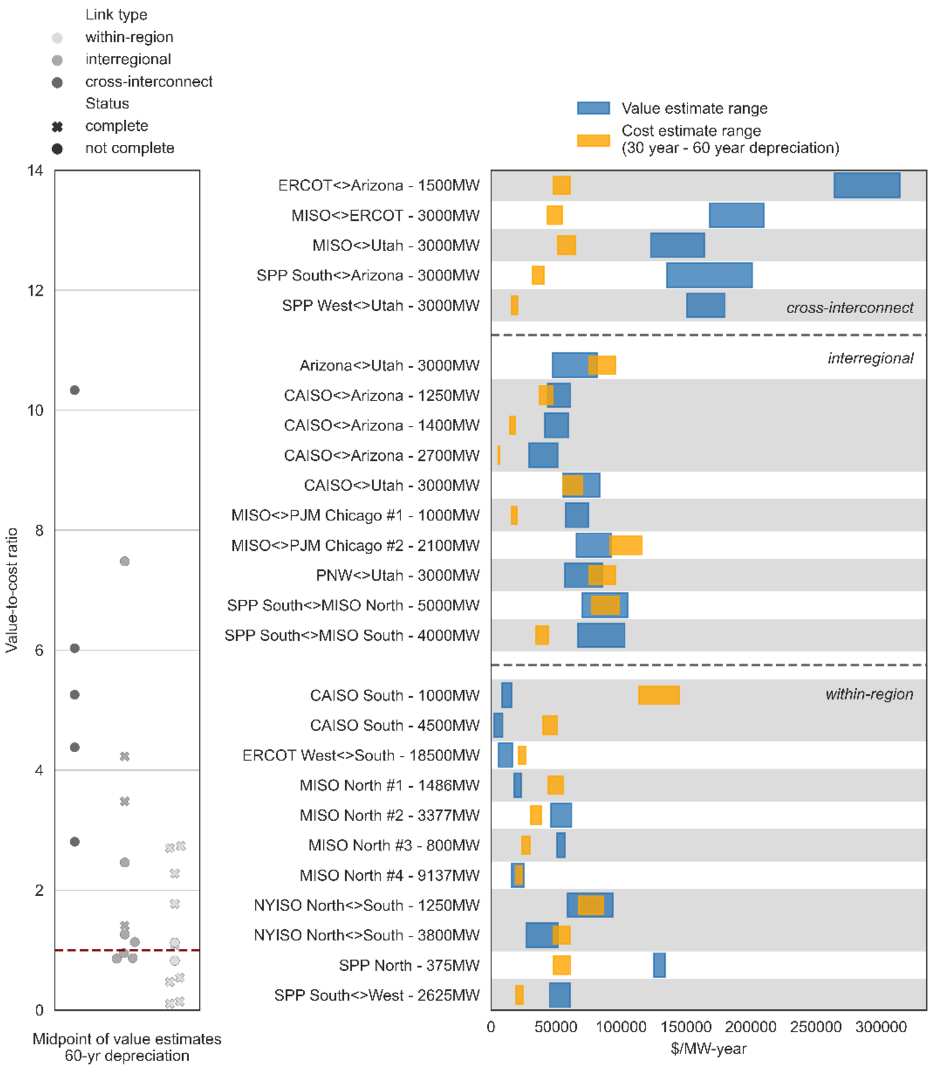

Supplementary Figure 7 Comparison of project costs to market value estimates that account for market depth <u>assuming the relevant market size is 50% of the size used in the main paper</u>. (left) Value-to-cost ratios where the value is the midpoint of the value estimate range in (right) and the cost estimate assumes 60-year depreciation. The horizontal dashed line at 1 represents the "break-even" ratio. (right) Range of market value (real-time) estimates accounting for market depth and annualized project cost estimates.

Supplemental Information about Methods

Algorithm 1 Transmission value estimates accounting for market depth based on supply curve models

Require: Non-decreasing supply curve functions: $\{f_\tau^s\}_{s\in\text{PriceNodes},\tau=1,2,\ldots,(\#\text{ of periods})}$

Require: Data on net load, prices, and transmission capacity for each hour and each link $a \leftrightarrow b$:

$DATA = \{(\text{NetLoad}_t^{Node\ a},\ \text{Price}_t^{Node\ a},\ \text{NetLoad}_t^{Node\ b}, \text{Price}_t^{Node\ b},\ \text{Transfer Capacity},\ \text{Period containing } t)\}$

Require: Price caps such that trade is assumed to be 0 if cap is met at both nodes: $\{cap^s\}_{s\in\text{PriceNodes}}$

Require: Parameters defining tolerance on residuals and for use in screening models:

Maximum acceptable absolute residual: Δ_1

Maximum acceptable multiple of the median absolute deviation from the median: Δ_2

Threshold for screening models (minimum proportion of points within acceptability buffer): Γ

▷ In this paper $\Delta_1 = \$25$, $\Delta_2 = 2$, and $\Gamma = 2/3$

$\{X_\tau^s\} = \{\{x_t^s \mid (x_t^s, \ldots, \tau^*) \in DATA\}_{\tau^*=1,2,\ldots,(\#\text{ of periods})}\}_{s\in\text{PriceNodes}}$ ▷ net load data

$\{Y_\tau^s\} = \{\{y_t^s \mid (\ldots, y_t^s, \ldots, \tau^*) \in DATA\}_{\tau^*=1,2,\ldots,(\#\text{ of periods})}\}_{s\in\text{PriceNodes}}$ ▷ price data

$\{T_s\}, \{P_s\} \leftarrow \textsc{ScreenModels}(\{f_\tau^s\}, \{(X,Y)_\tau^s\}, \Delta_1,\ \Delta_2,\ \Gamma)$ ▷ hours and periods with a usable model

$\{\tau_+^s,\ x_+^s,\ \tau_-^s,\ x_-^s\}_{s\in\text{PriceNodes}} \leftarrow \textsc{SteepestSlope}(\{f_\tau^s \mid \tau \in P_s\}, \{X_\tau^s \mid \tau \in P_s\})$

for all $(x_t^a, y_t^a, x_t^b, y_t^b, \ell, \tau) \in DATA$ **do**

 for $s \in \{a, b\}$ **do**

  $tol_\tau^s = \max\{\Delta_1,\ \Delta_2 \cdot \text{Median}_{y\in Y_\tau^s}\left(|y - \text{Median}_{y\in Y_\tau^s}(y)|\right)\}$

  if $t \in T_s$ **and** $|y_t^s - f_{s,\tau}(x_t^s)| \le tol_\tau^s$ **then**

   $\lambda_s = 0$

   $\delta_t^s = \text{argmin}_{\delta\in\mathbb{R}:\ f_\tau^s(\delta)=y_t^s} |x_t^s - \delta|$

   $g_t^s(z) := f_\tau^s\left((\delta_t^s + x_t^s)/2 - x_t^s + z\right) + y_t^s - f_\tau^s\left((\delta_t^s + x_t^s)/2\right)$ ▷ translated supply curve

   if $\min\{g_t^{s\prime}(z) \mid z \in [x_t^s - \ell,\ x_t^s + \ell]\} < 0$ **then** ▷ translated supply curve is decreasing near x_t^s

    $\lambda_s = 2$

    $h_t^s(z) := f_\tau^s(z) + y_t^s - f_\tau^s(x_t^s)$ ▷ vertically translated supply curve

    if $y_t^s \ge \text{Median}_{y\in Y_\tau^s}(y)$ **then** $g_t^s(z) := f_{\tau_+^s}^s(x_+^s - x_t^s + z) + y_t^s - f_{\tau_+^s}^s(x_+^s)$ ▷ translated steep supply curve

    else $g_t^s(z) := f_{\tau_-^s}^s(x_-^s - x_t^s + z) + y_t^s - f_{\tau_-^s}^s(x_-^s)$ ▷ translated steep supply curve

  else

   $\lambda_s = 1$

   if $y_t^s \ge \text{Median}_{y\in Y_\tau^s}(y)$ **then** $g_t^s(z) := f_{\tau_+^s}^s(x_+^s - x_t^s + z) + y_t^s - f_{\tau_+^s}^s(x_+^s)$ ▷ translated steep supply curve

   else $g_t^s(z) := f_{\tau_-^s}^s(x_-^s - x_t^s + z) + y_t^s - f_{\tau_-^s}^s(x_-^s)$ ▷ translated steep supply curve

   if $t \in T_a$ **then**

    $\lambda_s = 2$

    $h_t^s(z) := f_\tau^s(z) + y_t^s - f_\tau^s(x_t^s)$ ▷ vertically translated supply curve

 $c_t^{a,b}(z) := \mathbf{1}[y_t^a > y_t^b](g_t^a(x_t^a - z) - g_t^b(x_t^b + z)) + \mathbf{1}[y_t^a \le y_t^b](-g_t^a(x_t^a + z) + g_t^b(x_t^b - z))$

 $\ell' = \min\{z \mid c_t^{a,b}(z) = 0,\ z \in \mathbb{R}^+\}$ ▷ transfer capacity saturation point

 $v = \int_0^{\min\{\ell,\ell'\}} c_t^{a,b}(z)\, \mathrm{d}z$ ▷ transmission value estimate

 if $\lambda_a \in \{0,1\}$ **and** $\lambda_b \in \{0,1\}$ **then** $\underline{v}_t^{a,b} = \bar{v}_t^{a,b} = v$

 else

  if $\min\{\lambda_a, \lambda_b\} \le 1$ **then**

   if $\lambda_b == 2$ **then**

    $c_{t,2}^{a,b}(z) := \mathbf{1}[y_t^a > y_t^b](g_t^a(x_t^a - z) - h_t^b(x_t^b + z)) + \mathbf{1}[y_t^a \le y_t^b](-g_t^a(x_t^a + z) + h_t^b(x_t^b - z))$

   else

    $c_{t,2}^{a,b}(z) := \mathbf{1}[y_t^a > y_t^b](h_t^a(x_t^a - z) - g_t^b(x_t^b + z)) + \mathbf{1}[y_t^a \le y_t^b](-h_t^a(x_t^a + z) + g_t^b(x_t^b - z))$

  else

   $c_{t,2}^{a,b}(z) := \mathbf{1}[y_t^a > y_t^b](h_t^a(x_t^a - z) - h_t^b(x_t^b + z)) + \mathbf{1}[y_t^a \le y_t^b](-h_t^a(x_t^a + z) + h_t^b(x_t^b - z))$

  $\ell_2' = \min\{z \mid c_{t,2}^{a,b}(z) = 0,\ z \in \mathbb{R}^+\}$ ▷ transfer capacity saturation point

  $v_2 = \int_0^{\min\{\ell,\ell_2'\}} c_{t,2}^{a,b}(z)\, \mathrm{d}z$ ▷ transmission value estimate

  $\underline{v}_t^{a,b} = \min\{v, v_2\}$

  $\bar{v}_t^{a,b} = \max\{v, v_2\}$

 if $y_t^a = cap^a$ **and** $y_t^b = cap^b$ **then** $\underline{v}_t^{a,b} = \bar{v}_t^{a,b} = 0$

return $\bar{v}$, $\underline{v}$

Supplementary Algorithm 1 Transmission value estimates accounting for market depth based on supply curve models. $\overline{v}$ contains the higher value estimates and $\underline{v}$ contains the lower value estimates. $\mathbf{1}[\cdot]$ is the indicator function that evaluates to 1 if the operand is true and 0 if the operand is false.

Algorithm 2 Screen supply curve models to identify those with a poor fit

Require: Parameters defining size of the acceptability buffer:
  Maximum acceptable absolute residual: Δ_1
  Maximum acceptable multiple of the median absolute deviation from the median: Δ_2
Require: Threshold for minimum proportion of points within acceptability buffer: Γ
  ▷ In this paper $\Delta_1 = \$25$, $\Delta_2 = 2$, and $\Gamma = 2/3$
Require: Non-decreasing supply curve models: $\{f_\tau^s\}_{s \in \text{PriceNodes}, \tau=1,2,\ldots,(\#\text{ of periods})}$
Require: Data on net load and prices grouped by time period:
  $\{(X,Y)_\tau^s\} = \{\{(x_t^s, y_t^s)\}_{t=1,2,\ldots,\text{length of period }\tau}\}_{s \in \text{PriceNodes}, \tau=1,2,\ldots,(\#\text{ of periods})}$

procedure ScreenModels($\{f_\tau^s\}$, $\{(X,Y)_\tau^s\}$, Δ_1, Δ_2, Γ)
  for $s \in$ PriceNodes **do**
    $T_s = \emptyset$, $P_s = \emptyset$
    for $\tau = 1, 2, \ldots, (\#\text{ of periods})$ **do**
      $\bar{y} = \text{Median}_{y \in Y_\tau^s}(y)$
      $mad = \text{Median}_{y \in Y_\tau^s}(|y - \bar{y}|)$
      if $\Gamma |X_\tau^s| \le \Sigma_{(x_t, y_t) \in (X,Y)_\tau^s} \mathbf{1}[\, |y_t - f_\tau^s(x_t)| \le \max\{\Delta_1,\ \Delta_2 \cdot mad\}\,]$ **then**
        $P_s = P_s \cup \{\tau\}$
        $T_s = T_s \cup \{t \mid x_t^s \in X_\tau^s\}$
return $\{T_s\}_{s \in \text{PriceNodes}}$, $\{P_s\}_{s \in \text{PriceNodes}}$ ▷ hours and periods with a usable model

Supplementary Algorithm 2 Screen supply curve models to identify those with a poor fit

Algorithm 3 Identify the steepest supply curves at high and low net load levels

Require: Non-decreasing supply curve models: $\{f_\tau^s\}_{s \in \text{PriceNodes}, \tau=1,2,\ldots,(\#\text{ of periods})}$
Require: Net demand data partitioned by time period: $\{X_\tau^s\} = \{\{x_t^s\}_{t \in \tau}\}_{s \in \text{PriceNodes}, \tau=1,2,\ldots,(\#\text{ of periods})}$

procedure SteepestSlope($\{f_\tau^s\}$, $\{X_\tau^s\}$)
  for $s \in$ PriceNodes **do**
    $\tau_+^s = \text{argmax}_{\tau=1,2,\ldots,(\#\text{ of periods})}\{f_\tau^{s\prime}(z) \mid z = \max\{x \mid x \in X_\tau^s\}\}$
    $x_+^s = \max\{x \mid x \in X_{\tau_+^s}^s\}$
    $\tau_-^s = \text{argmax}_{\tau=1,2,\ldots,(\#\text{ of periods})}\{f_\tau^{s\prime}(z) \mid z = \min\{x \mid x \in X_\tau^s\}\}$
    $x_-^s = \min\{x \mid x \in X_{\tau_-^s}^s\}$
return $\{\tau_+^s,\ x_+^s,\ \tau_-^s,\ x_-^s\}_{s \in \text{PriceNodes}}$

Supplementary Algorithm 3 Identify the steepest supply curves at high and low net load levels

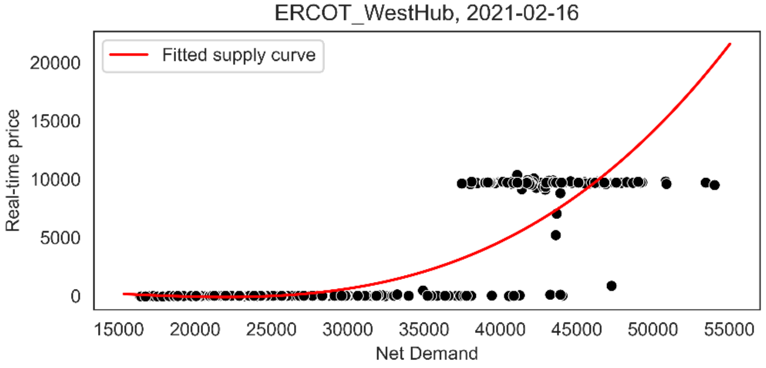

Supplementary Figure 8 Example of supply curve model that fails to pass the screening process established in Algorithm 2. Each point represents an hour for ERCOT's West Hub during 2021-02-16 to 2021-02-28, a period that overlaps Winter Storm Uri.

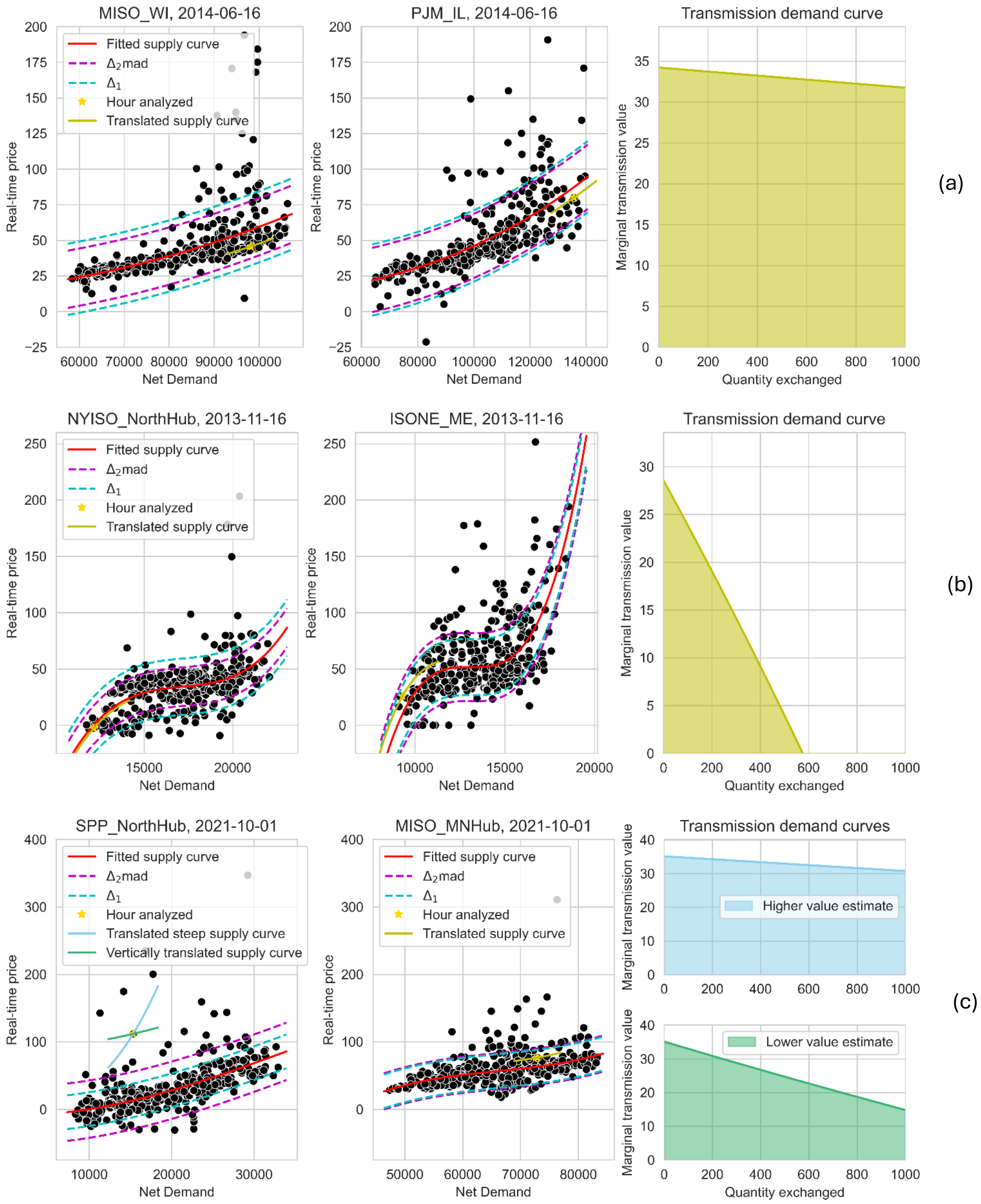

Supplementary Figure 9 Examples of methodology for estimating transmission market value accounting for depth of a 1000 MW transmission capacity increase. Each black circle represents one hour within a bi-monthly period. (a) Example with limited saturation effects. (b) Example with significant saturation effects such that <600 MW of the additional 1000 MW capacity available is transferred. (c) Example one node with a residual greater than the tolerance (dashed lines), so two transmission value estimates are calculated. The estimates use the same translated supply curve to represent MISO_MNHub, but different curves to represent SPP_NorthHub.

Supplementary Table 2 List of designated events used in the analysis, including NERC-identified periods of grid stress and key weather events identified in the literature.

Event Description	Start Date	End Date	#days	Sources
Cold	2/1/2011	2/4/2011	4	[4], [6]
--	4/4/2011	4/4/2011	1	[4]
Tornados, Severe Weather	4/27/2011	4/28/2011	2	[4]
--	6/30/2011	6/30/2011	1	[4]
--	7/1/2011	7/1/2011	1	[4]
Heat	7/19/2011	7/24/2011	6	[6]
Hurricane	8/25/2011	8/30/2011	6	[4], [6]
Southwest Blackout	9/8/2011	9/8/2011	1	[4]
Severe Weather Northeast Snowstorm	10/29/2011	10/29/2011	1	[4]
Severe Weather Tornadoes	3/2/2012	3/2/2012	1	[4]
Severe Thunderstorm	5/29/2012	5/29/2012	1	[4]
Heat (Novacheck), Thunderstorm Derecho (NERC SOR)	6/29/2012	7/7/2012	9	[4], [6]
Severe Thunderstorm	7/18/2012	7/18/2012	1	[4]
Severe Thunderstorm	7/24/2012	7/24/2012	1	[4]
Hurricane Isaac	8/28/2012	8/29/2012	2	[4]
Hurricane Sandy	10/29/2012	10/30/2012	2	[4]
Equipment Failure	2/8/2013	2/8/2013	1	[4]
Power System Condition, Fire	5/30/2013	5/30/2013	1	[4]
Severe Thunderstorms	6/13/2013	6/13/2013	1	[4]
Weather	6/23/2013	6/23/2013	1	[4]
Severe Weather, Fault and Equipment Failure	6/26/2013	6/27/2013	2	[4]
Rainfall Leading to Flooding, Severe Thunderstorms	7/8/2013	7/10/2013	3	[4]
Heatwave	9/9/2013	9/11/2013	3	[7]
Severe Ice & Snow Storm	11/17/2013	11/17/2013	1	[4]
Cold, Load Shed	12/4/2013	12/12/2013	9	[4], [6]
Polar Vortex	1/3/2014	1/10/2014	8	[3], [4]
Winterstorm	1/21/2014	1/24/2014	4	[4]
Winterstorm	1/29/2014	1/29/2014	1	[4]
Thunderstorms	7/8/2014	7/8/2014	1	[4]
Extreme Windstorm	12/11/2014	12/11/2014	1	[4]
Severe Winter Weather	1/8/2015	1/8/2015	1	[4]
Severe Winter Weather	2/20/2015	2/20/2015	1	[4]
Severe Weather	6/23/2015	6/23/2015	1	[4]
Severe Weather	6/30/2015	6/30/2015	1	[4]
Severe Weather	7/13/2015	7/13/2015	1	[4]
Severe Weather	7/18/2015	7/18/2015	1	[4]
Thunderstorm/Showers	7/20/2015	7/20/2015	1	[4]

Summer Weather	7/30/2015	7/30/2015	1	[4]
Excessive Rainfall, Thunder/Lightning Storm	10/23/2015	10/23/2015	1	[4]
Storm, Flooding, Straightline Winds	11/17/2015	11/17/2015	1	[4]
--	6/27/2016	6/28/2016	2	[4]
--	7/6/2016	7/6/2016	1	[4]
--	7/8/2016	7/8/2016	1	[4]
Severe weather	7/14/2016	7/14/2016	1	[4]
--	7/21/2016	7/21/2016	1	[4]
--	7/25/2016	7/25/2016	1	[4]
Severe weather	8/11/2016	8/11/2016	1	[4]
Severe weather	10/8/2016	10/8/2016	1	[4]
--	10/24/2016	10/24/2016	1	[4]
Winter storm	3/8/2017	3/8/2017	1	[4]
Wind storm	4/7/2017	4/7/2017	1	[4]
Unrelated coincidental generator outages	5/1/2017	5/1/2017	1	[4]
Hurricane Irma	9/11/2017	9/11/2017	1	[4]
Thomas Fire	12/4/2017	12/4/2017	1	[4]
Thomas Fire	12/10/2017	12/10/2017	1	[4]
Bomb Cyclone, Severe weather (load reduction), Severe weather (severe cold weather)	12/26/2017	1/19/2018	25	[3], [4]
Severe weather (winter NPCC, storm Riley)	3/2/2018	3/2/2018	1	[4]
Severe weather (late season snow, storm)	4/15/2018	4/15/2018	1	[4]
Severe weather (tornado, wind, hail)	5/15/2018	5/15/2018	1	[4]
Natchez Fire	8/11/2018	8/11/2018	1	[4]
Severe weather (hurricane Florence)	9/14/2018	9/14/2018	1	[4]
Severe weather (tropical storm Michael)	10/11/2018	10/11/2018	1	[4]
Severe weather (winter storm Avery)	11/15/2018	11/15/2018	1	[4]
Winter Storm Indra	1/21/2019	1/21/2019	1	[4]
Polar vortex 10idwest, Winter Storm Jayden	1/30/2019	2/1/2019	3	[3], [4]
Winter Storms Maya and Nadya	2/12/2019	2/12/2019	1	[4]
Wind Storm, Winter Storms Quiana and Ryan	2/24/2019	2/25/2019	2	[4]
Coincidental Generator Outages	7/22/2019	7/22/2019	1	[4]
Heat	8/5/2019	8/16/2019	12	[3]
Coincidental Generator Outages	9/3/2019	9/3/2019	1	[4]
Saddleridge Fire	10/11/2019	10/11/2019	1	[4]
Coincidental Generator Outages	11/27/2019	11/27/2019	1	[4]
Arctic outbreak + extreme cold + thunderstorms	1/11/2020	1/11/2020	1	[4]
Arctic outbreak + extreme cold + "Nor'easter"	1/12/2020	1/12/2020	1	[4]
Easter Tornado	4/13/2020	4/13/2020	1	[4]
Tropical Storm Amanda: Cristobal	6/3/2020	6/3/2020	1	[4]
Tropical Storm Amanda: Cristobal	6/9/2020	6/9/2020	1	[4]

Unrelated coincidental generator outages	7/1/2020	7/1/2020	1	[4]
Hurricane Isaias	8/4/2020	8/4/2020	1	[4]
Windstorms	8/10/2020	8/10/2020	1	[4]
CA/West heat, Extreme heat and demand with load shed-California	8/14/2020	8/19/2020	6	[3], [4]
Hurricane Laura	8/27/2020	8/27/2020	1	[4]
Johnson Fire	8/28/2020	8/28/2020	1	[4]
Wild fires	9/7/2020	9/8/2020	2	[4]
Ice storm + Hurricane Zeta	10/28/2020	10/28/2020	1	[4]
Hurricane Zeta	10/29/2020	10/29/2020	1	[4]
High winds	11/15/2020	11/15/2020	1	[4]
Texas cold	2/12/2021	2/20/2021	9	[3], [4]
Major thunderstorms	6/21/2021	6/21/2021	1	[4]
Heatwave, Heat Dome and major thunderstorms	6/26/2021	6/28/2021	3	[4]
Hurricane Nicholas and special protection system misoperation dropping generation	9/13/2021	9/13/2021	1	[4]
December windstorm and tornadoes	12/11/2021	12/11/2021	1	[4]
Winter Storm Elliott	12/22/2022	12/25/2022	4	[1], [2], [5]
Hurricane Ian	9/28/2022	9/30/2022	3	[5]
Hurricane Nicole	11/10/2022	11/11/2022	2	[5]
Southern Tornado Outbreak	3/30/2022	3/30/2022	1	[5]
Southeastern Tornado Outbreak	4/4/2022	4/6/2022	3	[5]
Kentucky and Missouri Flooding	7/26/2022	7/28/2022	3	[5]
Central Derecho	6/13/2022	6/13/2022	1	[5]
Southern and Central Severe Weather	5/1/2022	5/3/2022	3	[5]
Southern Severe Weather	4/11/2022	4/13/2022	3	[5]
Central Severe Weather	6/7/2022	6/8/2022	2	[5]
North Central Severe Weather	5/11/2022	5/12/2022	2	[5]
North Central and Eastern Severe Weather	7/22/2022	7/24/2022	3	[5]
Texas Hail Storms	2/21/2022	2/22/2022	2	[5]
North Central Hail Storms	5/9/2022	5/9/2022	1	[5]
North Central Hail Storms	5/19/2022	5/19/2022	1	[5]

Supplementary References

1. Energy Ventures Analysis. (2023) "Operation of the U.S. Power Generation Fleet During Winter Storm Elliott," https://www.evainc.com/wp-content/uploads/2023/02/2023_02_23-EVA-Winter-Storm-Elliott-Report.pdf
2. Goggin M. and Zimmerman Z., (2023) "The Value of Transmission During Winter Storm Elliott," Grid Strategies. https://acore.org/resources/the-value-of-transmission-during-winter-storm-elliott
3. Goggin M. (2021) "Transmission Makes the Power System Resilient to Extreme Weather," Grid Strategies. https://acore.org/transmission-makes-the-power-system-resilient-to-extreme-weather

4. NERC SOR (2022). “2022 State of Reliability, An Assessment of 2021 Bulk Power System Performance.” North American Electric Reliability Corporation. Reports for years 2012 – 2022 available at: https://www.nerc.com/pa/RAPA/PA/Pages/default.aspx
5. NOAA National Centers for Environmental Information (NCEI) U.S. Billion-Dollar Weather and Climate Disasters (2024). https://www.ncei.noaa.gov/access/billions/, DOI: 10.25921/stkw-7w73
6. Novacheck et al. (2021) “The Evolving Role of Extreme Weather Events in the US Power System with High Levels of Variable Renewable Energy” National Renewable Energy Lab. (NREL), NREL/TP-6A20-78394. https://doi.org/10.2172/1837959
7. PJM Interconnection (2013) “Technical Analysis of Operational Events and Market Impacts During the September 2013 Heat Wave.” https://hepg.hks.harvard.edu/sites/hwpi.harvard.edu/files/hepg/files/20131223-technical-analysis-of-operational-events-and-market-impacts-during-the-september-2013-heat-wave.pdf?m=1523366870
